# SSR2Marker: an integrated pipeline for identification of SSR markers within any two given genome-scale sequences

**DOI:** 10.1186/s43897-022-00033-0

**Published:** 2022-04-22

**Authors:** Junyang Yue, Yongsheng Liu

**Affiliations:** 1 School of Horticulture, Anhui Agricultural University, Hefei, 230036 China; 2 Agricultural Genomics Institute at Shenzhen, Chinese Academy of Agricultural Sciences, Shenzhen, 518124 China

## Introduction

Simple sequence repeats (SSRs), also known as microsatellites, are typically comprised of 1–6 nucleotide units repeated in tandem patterns (Ellegren 2004). Due to their high level of polymorphism, SSRs have become one of the most commonly used molecular markers for species identification, diversity assessment, linkage mapping, molecular breeding and QTL analysis (Vieira et al. 2016). Therefore, exploration and exploitation of SSR markers have attracted intense interests in plant breeding programs for the genotype and phenotype linkage analysis. However, traditionally experimental screening of SSR markers is extremely labor-intensive and time-consuming. With tremendously increasing volumes of high-throughput sequencing data, many bioinformatics tools have been proposed for automated SSR discovery and/or marker screening, but they have certain limitations in target sequence extraction, large-size data handling and/or overall performance (Supplementary Table 1).

Here, we report a novel pipeline, SSR2Marker, to specifically explore the candidate polymorphic SSR markers between any two given sequences at a large scale. It enables users to identify both monomorphic and dimorphic SSR markers for different purposes. Meanwhile, detailed information, including SSR motifs, primer pairs, amplified fragments, sequence sizes, length polymorphisms and statistics calculations, is also provided to facilitate subsequent genetic analyses and marker-assisted breeding. The source codes, examples and a complete manual of the SSR2Marker pipeline are freely available at https://github.com/aaranyue/SSR2Marker.

## Results

**Fig. 1:**
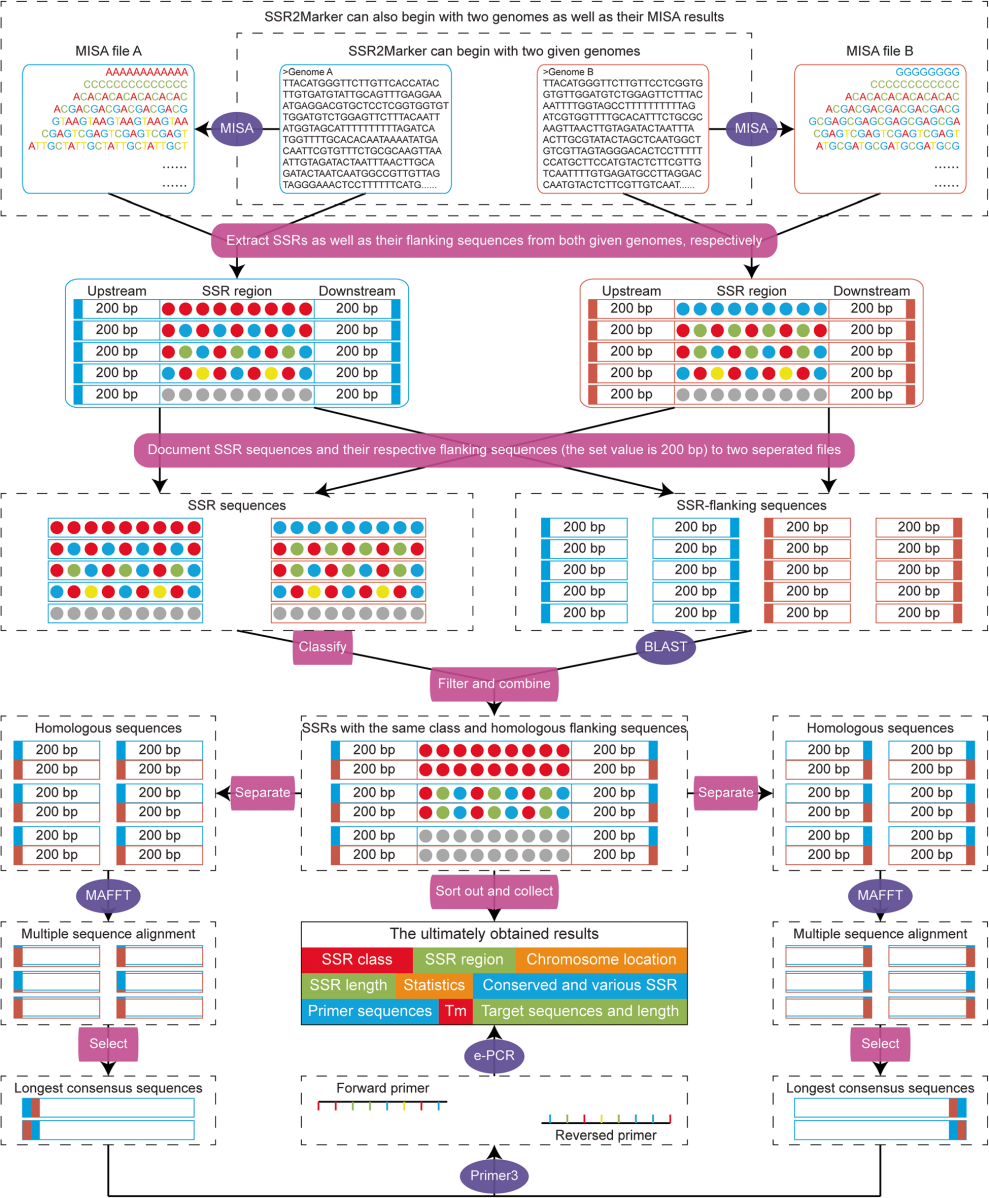
Schematic representation of the SSR2Marker pipeline. The detailed steps are described in the main text as well as in the manual released online

The SSR2Marker pipeline is designed as a local program consisting of the six main steps as follows (Fig. 1).

(i). Identification of SSR motifs: The required input data could be two DNA sequences from whatever source derived. SSR motifs as well as their corresponding information, such as SSR length, repeat class and number, are internally executed using MISA with slightly modified parameters (Thiel et al. 2003). If users already have the MISA results, they can also provide SSR information to run SSR2Marker program as a start point, which would save about one fifth of the computation time without losing the final reliability.

(ii). Comparison of repeat classes: By default, the basic nucleotide unit of SSR motif starts from the first nucleotide of the identified SSR marker (e.g., AAC is the nucleotide unit of AACAACAACAACAACAAC, while ACA is the nucleotide unit of ACAACAACAACAACAACA). As shown, frame shift and reverse complementary might cause diversity of the appearance of nucleotide units, which should be standardized for further processing. Here, a pre-prepared dictionary is employed to classify all possible nucleotide units into their representative classes, e.g., the AAC class = AAC, ACA, CAA, GTT, TGT and TTG. Only the SSR motifs within the same class are retained for further analysis.

(iii). Alignment of SSR-flanking regions: After obtaining the same SSR class, the flanking regions on both sides (upstream and downstream) of each SSR motif are extracted at a given length value (default setting is 200 bp). To reduce the influence of alignment biases that might be caused by SSR motifs, only the flanking regions are retrieved and aligned by using the BLAST program (*E*-value <= 1*e*-5, Identity > = 80). In consideration of inversion sequences, the defined upstream sequences are compared against not only themselves but also the downstream sequences, and vice versa. The desired SSR markers should simultaneously contain the alignments of upstream and downstream sequences from both FASTA files.

(iv). Selection of identical fragments: Even the alignments possess high homologies, there could be still numerous SNPs and/or gaps existed in the SSR-flanking regions, which could affect the ability of the PCR primers to anneal and eventually reduce the across-species transferability. Therefore, only the identical DNA fragments within the upstream and downstream sequences are retained with the assistance of MAFFT (Nakamura et al. 2018).

(v). Design of common primers: Among each SSR-flanking region, the longest identical DNA fragments of both sides are separately extracted and then jointly used as reference for the design of PCR primers using Primer3 (Untergasser et al. 2012). By default, a total of five primer pairs as well as their sequence length, Tm value and GC content are postulated. Finally, e-PCR is employed to determine the global specificity and sensitivity of primer annealing for each input sequence (Schuler 1997).

(vi). Classification of length polymorphism: Due to the high rates of DNA replication errors within SSR motifs, both the size of SSR repeats and the length of SSR-flanking regions show extensive intraspecies and interspecies variations. With the common primers in hand, it is easy to extract PCR amplicons from both of the input sequences. Based on the difference of amplicons in length, these SSR motifs can be classified into monomorphic (with length difference = 0) or dimorphic (with length difference > 0) markers.

Collectively, all these steps are implemented in the SSR2Marker pipeline written in Perl script. Only two separate files in FASTA format are required for the identification of SSR markers. When running, users can rapidly obtain the accurate and comprehensive information stored in several output files, such as monomorphism and dimorphism of SSR markers, length and location of SSR motifs, class and unit of SSR repeats, a maximum of five primer pairs with detailed parameters, amplified fragments by each pair of primers, and statistical results for every procedure during this pipeline.

For illustration purposes, we present a case study on the economically important kiwifruit cultivar ‘Jinyan’, which was bred from the interspecific hybridization between *Actinidia chinensis* and *Actinidia eriantha* (Zhong et al. 2012). After preparing the genome sequences of *A. chinensis* and *A. eriantha* (Yue et al. 2020; http://kiwifruitgenome.org/), only a single command line ‘perl SSR2Marker.pl -f1 Hongyang.fasta -f2 White.fasta’ is needed to start running the SSR2Marker pipeline on a Linux operating system (for details, a complete manual is provided online). This task took approximately 2.4 h on a Linux server, with 4 Gb memory and 2.50 GHz Dual-Core CPU. As a result, 1561 monomorphic and 13,733 dimorphic SSR markers are identified. Among these dimorphisms, only 2475 (~ 18.02%) are caused by the length variations of SSR motifs between *A. chinensis* and *A. eriantha*. On the contrary, the majority of dimorphic markers (11,258, ~ 81.98%) are resulted from length variations of SSR-flanking regions, which have often been routinely overlooked by most algorithms or simply considered as special cases.

## Discussion

We present SSR2Marker, a user-friendly pipeline for the identification of SSR markers and the design of primer pairs. By using the identical SSR-flanking regions as references, the designed primers are completely applicable to both of the two given sequences, exhibiting satisfactory performance in transferability rate and marker quantity. Meanwhile, SSR2Marker considers the polymorphic status of SSR markers based on sequence length differences of both SSR motifs and SSR-flanking regions, which have dramatically expanded the number of informative SSR markers. As shown in the case study of kiwifruit, 11,258 dimorphic markers are generated from the length variations in the SSR-flanking regions, accounting for ~ 81.98%. Furthermore, this pipeline is qualified for the detection of taxon-specific markers (i.e., monomorphic SSR markers). Taken together, SSR2Marker can help researchers to easily obtain plentiful SSR markers that will undoubtedly accelerate genetic studies on a wide range of organisms.

### Supplementary Information

**Additional file 1: Supplementary Table 1.** The major limitations of the currently available tools or databases developed for SSR marker analysis.

## Data Availability

All data generated or analyzed during this study are included in this published article.
