# Supplementary material for: SSR2Marker: an integrated pipeline for identification of SSR markers within any two given genome-scale sequences
Source: Mol Hortic. 2022 Apr 22;2:11. doi: 10.1186/s43897-022-00033-0 (PMC10515269; doi:10.1186/s43897-022-00033-0)
Supplement: Supplementary file 1 — Additional file 1: Supplementary Table 1. The major limitations of the currently available tools or databases developed for SSR marker analysis. [file 43897_2022_33_MOESM1_ESM.docx]

**Supplementary Table 1**. The major limitations of the currently available tools or databases developed for SSR marker analysis.

| **Major limitations** | **Designed just for the identification of SSR motifs** | **Integrated with primer design but solely confined to single sequence** | **Dependent on the transferability of primer pairs from the reference genome** | **Incapable of dealing with big data** | **Missing statistical analyses** | **Limited information focusing on a certain species or genus** | **Out-of-date content** | **Reference** |
| --- | --- | --- | --- | --- | --- | --- | --- | --- |
| MISA | + |  |  |  | + |  |  | Thiel et al. 2003 |
| BatchPrimer3 |  | + |  |  | + |  |  | You et al. 2008 |
| CandiSSR |  |  | + |  | + |  |  | Xia et al. 2016 |
| GMATA |  |  | + |  |  |  |  | Wang et al. 2016 |
| IDSSR |  |  | + |  |  |  |  | Guang et al. 2019 |
| Kmer-SSR | + |  |  |  |  |  |  | Pickett et al. 2017 |
| MSDB |  |  |  |  |  | + | + | Avvaru et al. 2017 |
| PolyMorphPredict |  |  | + |  |  |  |  | Das et al. 2019 |
| PolySSR |  |  |  | + |  |  |  | Duran et al. 2013 |
| pSTR Finder |  |  |  |  | + |  |  | Lee et al. 2015 |
| QDD |  | + |  |  |  |  |  | Meglécz et al. 2014 |
| SSR Locator |  | + |  | + |  |  |  | da Maia et al. 2008 |
| SSRPoly |  |  |  | + |  |  |  | Tang et al. 2008 |
| VigSatDB |  |  |  |  |  | + | + | Jasrotia et al. 2019 |

**References**

Avvaru AK, Saxena S, Sowpati DT, et al. MSDB: a comprehensive database of simple sequence repeats. Genome Biol. Evol. 2017;9:1797-802.

da Maia LC, Palmieri DA, de Souza VQ, et al. SSR Locator: tool for simple sequence repeat discovery integrated with primer design and PCR simulation. Int. J. Plant Genomics. 2008;1:412696.

Das R, Arora V, Jaiswal S, et al. PolyMorphPredict: A universal web-tool for rapid polymorphic microsatellite marker discovery from whole genome and transcriptome data. Front. Plant Sci. 2019;9:1966.

Duran C, Singhania R, Raman H, et al. Predicting polymorphic EST-SSRs *in silico*. Mol. Ecol. Resour. 2013;13:538-45.

Guang XM, Xia JQ, Lin JQ, et al. IDSSR: An eﬃcient pipeline for identifying polymorphic microsatellites from a single genome sequence. Int. J. Mol. Sci. 2019;20:3497.

Jasrotia RS, Yadav PK, Iquebal MA, et al. VigSatDB: genome-wide microsatellite DNA marker database of three species of *Vigna* for germplasm characterization and improvement. Database (Oxford). 2019;1:baz055.

Lee JC, Tseng B, Ho BC, et al. pSTR Finder: a rapid method to discover polymorphic short tandem repeat markers from whole-genome sequences. Investig. Genet. 2015;6:10.

Meglécz E, Pech N, Gilles A, et al. QDD version 3.1: A user-friendly computer program for microsatellite selection and primer design revisited: Experimental validation of variables determining genotyping success rate. Mol. Ecol. Resour. 2014;14:1302-13.

Pickett BD, Miller JB, Ridge PG. Kmer-SSR: A fast and exhaustive SSR search algorithm. Bioinformatics. 2017;33:3922-8.

Tang J, Baldwin SJ, Jacobs JM, et al. Large-scale identiﬁcation of polymorphic microsatellites using an *in silico* approach. BMC Bioinform. 2008;9:374.

Thiel T, Michalek W, Varshney RK, et al. Exploiting EST databases for the development and characterization of gene-derived SSR-markers in barley (*Hordeum vulgare* L.). Theor. Appl. Genet. 2003;106:411-22.

Wang X, Wang L. GMATA: An integrated software package for genome-scale SSR mining, marker development and viewing. Front. Plant Sci. 2016;7:1350.

Xia EH, Yao QY, Zhang HB, et al. CandiSSR: An efficient pipeline used for identifying candidate polymorphic SSRs based on multiple assembled sequences. Front. Plant Sci. 2016;6:1171.

You FM, Huo N, Gu YQ, et al. BatchPrimer3: a high throughput web application for PCR and sequencing primer design. BMC Bioinform. 2008;9:253.
